# Human RTEL1 associates with Poldip3 to facilitate responses to replication stress and R-loop resolution

**DOI:** 10.1101/gad.330050.119

**Published:** 2020-08-01

**Authors:** Andrea Björkman, Søren L. Johansen, Lin Lin, Mike Schertzer, Dimitris C. Kanellis, Anna-Maria Katsori, Søren T. Christensen, Yonglun Luo, Jens S. Andersen, Simon J. Elsässer, Arturo Londono-Vallejo, Jiri Bartek, Kenneth B. Schou

**Affiliations:** 1 Division of Genome Biology, Department of Medical Biochemistry and Biophysics, Science for Life Laboratory, Karolinska Institute, Solna 171 77, Sweden;; 2 Department of Cell Biology and Physiology, University of Copenhagen, DK-2100 Copenhagen, Denmark;; 3 Department of Biomedicine, Aarhus University, Aarhus 8200, Denmark;; 4 Steno Diabetes Center Aarhus, Aarhus University Hospital, Aarhus 8200, Denmark;; 5 3UMR 3244 (Telomere and Cancer Laboratory), Centre National de la Recherche Scientifique, Institut Curie, PSL Research University, Sorbonne Universités, Paris 75005, France;; 6 Department of Biochemistry and Molecular Biology, University of Southern Denmark, DK-5230 Odense M, Denmark;; 7 Danish Cancer Society Research Centre, DK-2100 Copenhagen, Denmark

**Keywords:** RTEL1, helicase, R-loop, DNA:RNA hybrid, Poldip3, POLδ, polymerase δ, DNA repair, DNA damage response, telomere maintenance, dyskeratosis congenita, Hoyeral-Hreiderson syndrome

## Abstract

RTEL1 helicase is a component of DNA repair and telomere maintenance machineries. While RTEL1's role in DNA replication is emerging, how RTEL1 preserves genomic stability during replication remains elusive. Here we used a range of proteomic, biochemical, cell, and molecular biology and gene editing approaches to provide further insights into potential role(s) of RTEL1 in DNA replication and genome integrity maintenance. Our results from complementary human cell culture models established that RTEL1 and the Polδ subunit Poldip3 form a complex and are/function mutually dependent in chromatin binding after replication stress. Loss of RTEL1 and Poldip3 leads to marked R-loop accumulation that is confined to sites of active replication, enhances endogenous replication stress, and fuels ensuing genomic instability. The impact of depleting RTEL1 and Poldip3 is epistatic, consistent with our proposed concept of these two proteins operating in a shared pathway involved in DNA replication control under stress conditions. Overall, our data highlight a previously unsuspected role of RTEL1 and Poldip3 in R-loop suppression at genomic regions where transcription and replication intersect, with implications for human diseases including cancer.

## Abstract (precis)

In this study from Björkman et al., the authors sought to understand how RTEL1 helicase preserves genomic stability during replication. They demonstrate that RTEL1 and the Polδ subunit Poldip3 form a complex and are mutually dependent in chromatin binding after replication stress, and loss of RTEL1 and Poldip3 leads to marked R-loop accumulation that is confined to sites of active replication, thus highlighting a previously unknown role of RTEL1 and Poldip3 in R-loop suppression at genomic regions where transcription and replication intersect.

RTEL1 (regulator of telomere length 1) was identified as a cancer susceptibility gene (Shete et al. 2009; Wrensch et al. 2009; Vannier et al. 2014) and implicated in a number of telomere dysfunction syndromes (Ballew et al. 2013a,b; Deng et al. 2013; Le Guen et al. 2013; Walne et al. 2013). Studies in both mice and human cells have shown that RTEL1 plays dual roles in preserving both telomere integrity and prevention of chromosomal abnormalities on a genome-wide scale (Ding et al. 2004; Vannier et al. 2013; Speckmann et al. 2017; Porreca et al. 2018). Accordingly, RTEL1 was previously demonstrated to act as an antirecombinase by evading excessive sister chromatid exchange and resolving DNA D-loop recombination intermediates and quadruplex structures during DNA repair and meiotic crossover (Barber et al. 2008; Youds et al. 2010; Vannier et al. 2012). More recently, RTEL1 was shown to play a broader role in replication through association with the proliferating cell nuclear antigen (PCNA) of the replisome, pointing to a critical role of RTEL1 at the replication fork for genetic stability (Vannier et al. 2013). However, whether PCNA represents the key physiological partner for the function(s) of RTEL1 in DNA replication remains to be addressed. Likewise, the localization and function of RTEL1 at the replisome remains unclear.

In this study, we examined the emerging role of RTEL1 in DNA replication. We identified a functional interplay between RTEL1 and the Poldip3 protein. Poldip3 has putative functions in two different complexes in human cells, namely the DNA polymerase δ (POLδ) and the TREX complex, respectively, implicating Poldip3 in diverse biological processes including DNA synthesis and mRNA trafficking. We found that RTEL1 and Poldip3 physically interact and recruit to chromatin in a mutually dependent manner in response to replication stress. Consistently, RTEL1 and Poldip3 depletion leads to elevated RNA–DNA hybrid (R-loop) accumulation and these R-loops are confined to sites of active replication. Furthermore, we show here that the R-loop accumulation after RTEL1 and Poldip3 depletion occurs at genomic common fragile sites, rDNA and telomeres. Our results reveal a new role of RTEL1 (and Poldip3) in protection against genome instability by preventing excessive R-loop accumulation after replication stress, a condition emerging as a hallmark of cancer and implicated in aging (Halazonetis et al. 2008; Merchut-Maya et al. 2019), thus providing mechanistic insights into the role of RTEL1 in safeguarding proper genome-wide replication and genomic integrity.

## Results

### Poldip3 is a novel RTEL1-associated protein

**Figure 1.**
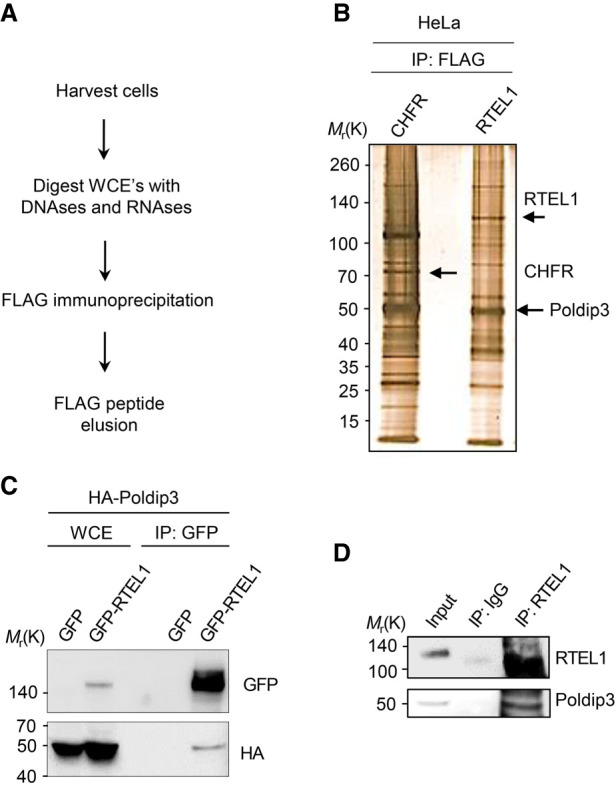
RTEL1 associates with Poldip3. (*A*) Schematic flow chart of the Flag affinity purification protocol of Flag-RTEL1. Flag-CHFR or Flag-RTEL1 or expressing HeLa cells were harvested and whole cell lysates were treated with a cocktail of DNases and RNases. Flag-RTEL1 fusion proteins were Flag immunopurified, eluted with Flag peptides, and immunocomplexes resolved by PAGE. (*B*) Silver staining of resolved proteins from the Flag-RTEL1 immunoprecipitation. Control immunoprecipitation was performed with a Flag-CHFR fusion protein. CHFR was used as control since it, like RTEL1, is a RING finger-containing protein. Flag-CHFR, Flag-RTEL1 and Poldip3 are indicated with arrows. (*C*) GFP-RTEL1 binds HA-Poldip3. HEK293T cells were cotransfected with HA-Poldip3 and either GFP or GFP-RTEL1 and incubated for 24 h prior to harvest. Extracts were subjected to GFP immunoprecipitation followed by immunoblotting with HA and GFP antibodies. (*D*) Endogenous RTEL1 binds endogenous Poldip3. U2OS cells were harvested in EBC buffer and cell lysates were treated as in *A.* Precleared lysates were subjected to immunoprecipitation with either IgG or RTEL1 antibody. RTEL1 immunocomplexes were released from the RTEL1-conjugated resin by treatment with glycine-HCl buffer.

To explore the function of RTEL1 in the regulation of cell proliferation and DNA repair, we searched for novel RTEL1-interacting proteins using the Flag tag affinity purification method. To enrich crude lysates for nuclear proteins including those proteins enriched on chromatin, we digested whole-cell extracts with a panel of DNA and RNA nucleases prior to Flag affinity purification analysis (Fig. 1A). The Flag-purified protein complexes were resolved by gel electrophoresis and proteins were visualized by silver staining (Fig. 1B). Flag-RTEL1 protein complexes revealed several specifically coeluted proteins, one of which was identified by mass spectrometry as the PDIP46/Poldip3/SKAR (subsequently referred to as Poldip3) (Supplemental Fig. S1A), a poorly understood protein with dual roles in the nuclear mRNA trafficking TREX/THO complex and DNA polymerase δ (Polδ) complex (Heath et al. 2016; Lee et al. 2017). We confirmed this interaction by coimmunoprecipitation where GFP-tagged RTEL1 specifically pulled down HA-tagged Poldip3 when expressed in human HEK293T cells (Fig. 1C). This interaction was not mediated by unspecific DNA intermediates since DNA nuclease treatment of whole-cell lysates did not affect the interaction (Supplemental Fig. S1B). In addition, we could demonstrate that endogenous RTEL1 and Poldip3 proteins interacted in U2OS cells (Fig. 1D), suggesting a biologically relevant function of this interplay.

**Figure 2.**
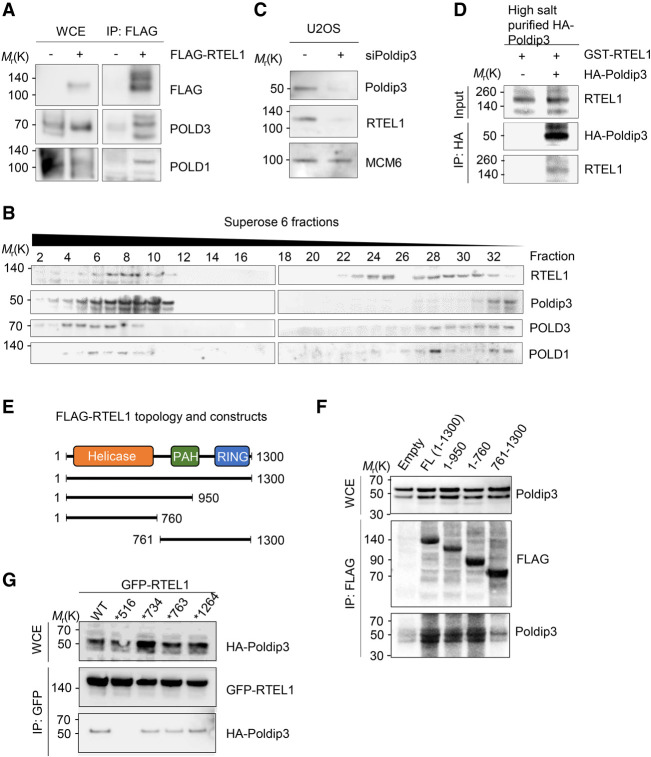
RTEL1 binds other Poldδ subunits and directly to Poldip3 through its helicase domain. (*A*) RTEL1 binds POLD3 and POLD1 subunits of the Polδ complex. Cell extracts from HEK293T cells transfected with Flag-RTEL1 or mock transfected were cleared and subjected to Flag immunoprecipitation with anti-Flag beads followed by PAGE. Proteins were probed by immunoblotting using the indicted antibodies. (*B*) Gel filtration analysis of HEK293T cell extracts. Proteins were probed by immunoblotting using the indicted antibodies. (*C*) Codepletion of RTEL1 by Poldip3 silencing. U2OS cells transfected with either control or Poldip3 siRNAs were incubated 4 d and subsequently harvested and proteins immunoblotted using the indicated antibodies. (*D*) Purified RTEL1 and Poldip3 interact in vitro. Bacterially purified GST-RTEL1 was incubated with high-salt-purified HA (control) or HA-Poldip3 on HA-conjugated beads. Eluded protein complexes were subjected to immunoblot analysis and proteins were probed using either HA or RTEL1 antibodies. (*E*) Schematic representation of Flag-RTEL1 deletion constructs. (*F*) RTEL1 binds Poldip3 primarily via its 4FeS helicase domain. HEK293T cells were transfected with Flag-RTEL1 full-length or deletion constructs spanning the indicated regions of RTEL1 to determine the Poldip3-binding site. The cell lysates were subjected to Flag immunoprecipitation followed by 3xFlag peptide elution and, subsequently, proteins were subjected to immunoblotting with Flag or Poldip3 antibodies. (*G*) GFP pull-down analysis of mutated GFP-RTEL1. HEK293T cells were cotransfected with HA-Poldip3 and either GFP-RTEL1 WT or GFP-RTEL1 containing the Hoyeraal-Hreidarsson syndrome mutations at M516I, L734R, G763V, or R1264H. Cell extracts were subjected to GFP immunoprecipitation followed by immunoblotting with GFP or HA antibodies.

Given that Poldip3 was recently demonstrated to be a stoichiometric subunit of the DNA polymerase δ (POLδ) holocomplex (Lee et al. 2017) and additional POLδ complex subunits have previously been identified in RTEL1 immunocomplexes (i.e., POLD1 and POLD3) (Vannier et al. 2013; Schertzer et al. 2015), we asked whether RTEL1 might bind additional components of the POLδ holocomplex. Indeed, besides binding to Poldip3, coimmunoprecipitation assays also identified specific physical interactions between RTEL1 and POLD1 and POLD3 (Fig. 2A). Supporting this finding, upon fractionation of whole-cell extracts by size exclusion chromatography, RTEL1 cofractionated with Poldip3 as well as POLD1 and POLD3 subunits (Fig. 2B) thus establishing that RTEL1 and the POLδ holoenzyme reside in a genuine protein complex. Since the evidence suggested that RTEL1 and Poldip3 might exist in a stable complex, we assessed whether their respective protein stabilities might be affected by each other, with immunoblot analyses of RTEL1 and Poldip3 in U2OS cells that had been depleted of Poldip3 using RNA interference. RTEL1 protein levels were dramatically decreased in U2OS cells depleted of Poldip3 (Fig. 2C), further supporting the notion that RTEL1 and Poldip3 associate in a stable complex. As RTEL1 also interacts with PCNA (Vannier et al. 2013), similarly to the replisome component POLδ, we examined whether RTEL1 interacts directly with Poldip3. High-salt-purified HA-Poldip3 coupled to an anti-HA resin was examined for its capacity to bind to bacterially purified GST-tagged RTEL1 or GST alone. A HA pull-down assay revealed that HA-Poldip3 bound specifically to GST-RTEL1, indicating that RTEL1 and Poldip3 interact directly (Fig. 2D). To gain further insight into the structural requirements for the RTEL1–Poldip3 interaction, we performed a deletion analysis to delineate the minimal region of RTEL1 required for its interaction with Poldip3 (Fig. 2E). Truncation of RTEL1 indicated that Poldip3 bound prominently to the RTEL1 N terminus harboring the helicase domain (Fig. 2F), further supporting the notion that RTEL1 and Poldip3 reside in a physical complex. Considering that disease-associated mutations in *RTEL1* previously associated with dyskeratosis congenita/Hoyeraal-Hreidarsson syndrome are scattered throughout the tertiary structure of RTEL1, including some in the helicase domain, we asked whether any of these mutations might disrupt the integrity of the emerging RTEL1–Poldip3 complex (Supplemental Fig. S2A). Interestingly, among the mutated versions of RTEL1, RTEL1 containing a methionine-to-isoleucine substitution at position 516 (M516I) demonstrated a dramatically reduced capacity to bind Poldip3 (Fig. 2G; Supplemental Fig. S2B). Methionine 516 is a highly conserved residue in the helicase domain of RTEL1 (Supplemental Fig. S2B), suggesting that the structural integrity of the helicase domain is critical for RTEL1 binding to Poldip3.

### RTEL1 and Poldip3 recruit to chromatin after topoisomerase I inhibition

**Figure 3.**
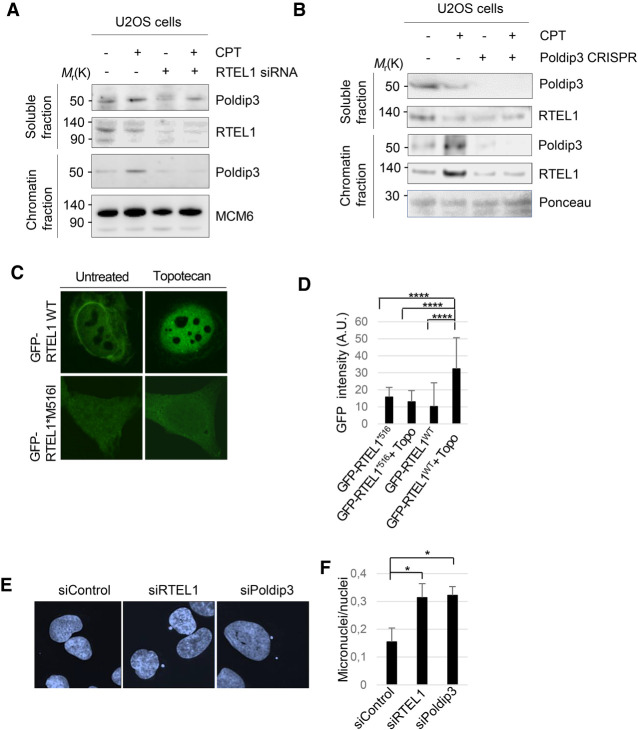
RTEL1 and Poldip3 bind chromatin in a mutually dependent manner after CPT treatment. (*A*) RTEL1 is required for chromatin recruitment of Poldip3 after CPT treatment. Whole cell extracts of WT or RTEL1 silenced U2OS cells were treated with CPT for 24 h, harvested, and split into soluble and chromatin fractions and chromatin bound proteins were released by acid extraction. Proteins were detected by immunoblotting with the indicated antibodies. (*B*) Poldip3 is required for chromatin recruitment of RTEL1 after CPT treatment. Whole-cell extracts of WT or Poldip3 CRISPR silenced U2OS cells were treated with CPT for 24 h, harvested, and split into soluble and chromatin fractions and chromatin-bound proteins were released by acid extraction. Proteins were detected by immunoblotting with the indicated antibodies. (*C*) GFP-RTEL1 WT or M516I mutated GFP-RTEL1 nuclear localization. U2OS cells stably expressing GFP-RTEL1 WT or M516 mutated GFP-RTEL1 were left untreated or treated with topotecan for 24 h. (*D*) Quantification of nuclear localization of GFP-RTEL1in U2OS cells stably expressing GFP-RTEL1 WT or GFP-RTEL1*M516I. Nuclear minus cytoplasmic staining is shown. (****) *P* < 0.0001, two-tailed Mann-Whitney test. (*E*) Increased micronuclei formation in cells depleted of RTEL1 or Poldip3. Representative images showing DAPI staining in U2OS cells treated with control, RTEL1, or Poldip3 siRNA. (*F*) Quantification of micronuclei in U2OS cells treated with control, RTEL1, or Poldip3 siRNAs. Mean and SD of three independent experiments are shown. (*) *P* < 0.05, Student's *t*-test.

Because RTEL1 function has been implicated in DNA replication (Vannier et al. 2013), analogous to POLδ, we investigated the possible regulatory role of RTEL1 toward Poldip3 functions in the DNA damage response upon replication stress. Exposure to replication inhibitors hydroxyurea (HU) or camptothecin (CPT) failed to augment the association of ectopically expressed RTEL1 and Poldip3 (Supplemental Fig. S2C), suggesting that the RTEL1–Poldip3 complex stability is unaffected by replication stress. RTEL1- and Poldip3-deficient cells were previously shown to be hypersensitive to topoisomerase I (TOPOI) poisons (Barber et al. 2008; Tumini et al. 2016). This prompted us to test whether RTEL1 is involved in the chromatin loading of Poldip3 in TOPOI-inhibited cells in S phase. Interestingly, while TOPOI inhibition by CPT treatment increased chromatin retention of Poldip3 in RTEL1-proficient cells, RTEL1 ablation markedly impaired this outcome (Fig. 3A), indicating that RTEL1 is involved in chromatin accumulation of Poldip3 after TOPOI inhibitor-induced replication fork stalling. Because RTEL1 and Poldip3 reside in a stable complex, we assessed whether Poldip3 depletion might reciprocally compromise RTEL1 accumulation on chromatin after TOPOI inhibition. Indeed, whereas Poldip3 silencing by clustered regularly interspaced short palindromic repeats (CRISPR/Cas9) recapitulated the RTEL1 protein reduction observed with Poldip3 siRNA treatment (Fig. 2C), residual RTEL1 protein showed reduced recruitment to chromatin after CPT treatment (Fig. 3B), indicating that RTEL1 and Poldip3 act together on chromatin in response to TOPOI inhibition-induced replication stress. Supporting the notion that RTEL1 function relies on its interaction with Poldip3, we found that GFP-tagged RTEL1 wild type but not M516I mutated RTEL1 translocated from the cytoplasm to the nucleus after TOPOI inhibition (Fig. 3C,D). A key phenotype of RTEL1 deficiency is hyperrecombination, reflected in an increase of RAD51 nuclear foci due to the accumulation of recombination intermediates that persist and fail to be appropriately repaired (Barber et al. 2008). Likewise, we found that Poldip3 loss augmented nuclear RAD51 staining (Supplemental Fig. S2D,E), indicating that Poldip3 deficiency phenocopies the hyperrecombination phenotype of RTEL1. Consistent with the occurrence of accumulated recombination intermediates, the silencing of either RTEL1 or Poldip3 resulted in increased chromosomal instability as evident from the marked increase of micronuclei (Fig. 3E,F). The increased genomic instability was also apparent from the increase in the nuclear staining of γH2A.X, a marker of DNA damage, upon RTEL1 or Poldip3 loss (Supplemental Fig. S3A,B). These results indicate that the loss of either protein leads to persistent DNA damage. Hence, these findings further support the notion that RTEL1 and Poldip3 exert their functions on chromatin in a mutually dependent manner.

### Poldip3 and RTEL1 suppress R-loop accumulation

**Figure 4.**
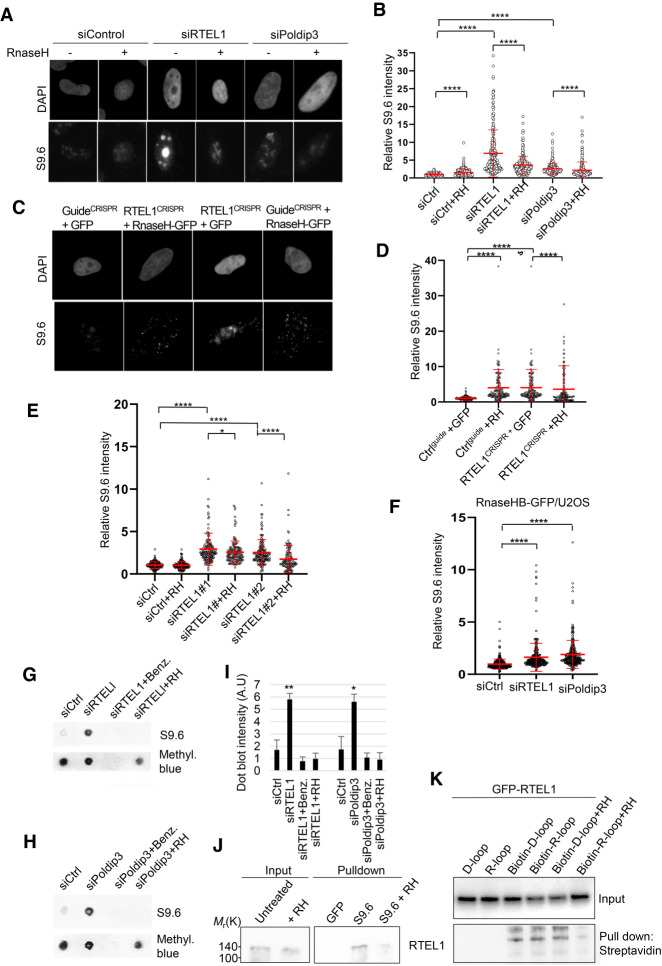
Increased accumulation of R loops in RTEL1- or Poldip3-depleted cells. (*A*) Increased accumulation of R loops in cells depleted of RTEL1 or Poldip3. Representative images of DAPI and S9.6 fluorescence staining in U2OS cells treated with control, RTEL1, or Poldip3 siRNAs. (*B*) Quantification of nuclear S9.6 fluorescence intensity in U2OS cells treated with control, RTEL1, or Poldip3 siRNA. Cells conditionally overexpressing GFP-RNase H (RH) with doxycline were used. (*C*) Increased amount of R-loops in RTEL1 knockout U2OS cells. U2OS cells transiently transfected with CRISPR–CAS9 guides against RTEL1 or control guide and RNase H or GFP. (*D*) Quantification of nuclear S9.6 fluorescence intensity in U2OS cells transiently transfected with CRISPR-CAS9 guides against RTEL1 or control guide and RNase H or GFP. (*E*) Increased accumulation of R-loops in U2OS cells depleted of RTEL1 using two different RTEL1 siRNAs. Quantification of nuclear S9.6 fluorescence intensity in U2OS cells conditionally overexpressing GFP-RNase H (RH) treated with control or RTEL1 siRNAs. (*F*) Increased GFP-RNase H/R-loop binding domain recruitment to nucleus in cells depleted of RTEL1 or Poldip3. Quantification of GFP fluorescence intensity in U2OS-RNase HB-GFP cells treated with control, RTEL1, or Poldip3 siRNA. (*G*) R-loop dot blot analysis. U2OS cells were treated with either control, RTEL1, or Poldip3 siRNA followed by a 3-d incubation. Genomic DNA was isolated from cell lysates, bulk RNA digested with RNase A, and DNA was subsequently hand spotted on activated nylon membranes as indicated. DNA digested with the nuclease benzonase (Benz.) or RNase H (RH) was used as a control. (*H*) R-loop dot blot analysis. U2OS cells were treated with either control or Poldip3 siRNA followed by a 3-d incubation. Genomic DNA was isolated from cell lysates, bulk RNA digested with RNase A, and DNA was subsequently hand-spotted on activated nylon membranes as indicated. DNA digested with the nuclease benzonase (Benz.) or RNase H (RH) was used as a control. (*I*) Quantification of *G* and *H*. Mean and SD are shown. (*) *P* < 0.05; (**) *P* < 0.01, Student's *t*-test. (*J*) RTEL1 binds to R-loops. U2OS cells left untreated or treated with CPT for 24 h were harvested and R-loops were immunoprecipitated from cleared nuclear extracts with the S9.6 antibody. Nuclear extracts from cells treated with RNase H (RH) was used as a control. (*K*) RTEL1 interacts with R-loops and D-loops in vitro. Extracts of U2OS cells stably expressing GFP-RTEL1 WT were incubated with biotin-coupled R-loop or D-loop hybrids immobilized on streptavidin beads, and GFP-RTEL1 was immunoblotted with GFP antibody. Cell extracts incubated with purified RNase H (RH) was used as a control. Mean and SD are plotted. (*B*,*D*,*E*,*F*) (*) *P* < 0.05; (****) *P* < 0.0001, two-tailed Mann-Whitney test. Data from three or more independent experiments are combined and immunofluorescence intensities are normalized to siCtrl.

Besides its presence in the POLδ complex, Poldip3 is also a putative component of THO/TREX, a complex implicated in several steps of nuclear mRNP biogenesis, including transcription, 3′ end processing, and export (Lee et al. 2017). Notably, THO/TREX disruption is associated with the accumulation of R-loops; i.e., discrete RNA:DNA hybrids that are formidable barriers to the replication fork progression (Heath et al. 2016). Furthermore, DinG, RTEL1's ancient ortholog in bacteria, has been reported to resolve R loop structures (Boubakri et al. 2010). To investigate whether Poldip3 or RTEL1 deficiency leads to excess R-loop accumulation, we examined Poldip3 and RTEL1 silenced cells for basal levels of R-loop formation by immunofluorescence microscopy with the widely used S9.6 antibody recognizing DNA:RNA hybrids. To exclude the possibility that the S9.6 antibody detects RNA species different from R-loops, we assessed R-loops after RTEL1 and Poldip3 depletion in cells generated to conditionally overexpress the R-loop-specific degrading nuclease RNase H. U2OS cells deficient for Poldip3 exhibited elevated R-loops compared with control cells (Fig. 4A,B; Supplemental Fig. S3C). As with Poldip3-deficient cells, cells depleted for RTEL1 by siRNA treatment displayed increased R-loop accumulation as compared with control siRNA-treated cells (Fig. 4A,B; Supplemental Fig. S3D). The S9.6 signal was particularly strong in the nucleolus, a region prone to R-loop accumulation due to highly active transcription of repetitive rDNA (El Hage et al. 2010; Stuckey et al. 2015). Interestingly, doxycycline-induced expression of RNase H effectively eliminated the R-loop accumulation after RTEL1 and Poldip3 depletion (Fig. 4A,B), indicating that the R-loop accumulation after depletion of either protein is specific. The increase in S9.6 staining in control cells treated with RNase H could be due to the stress induced by RNase H overexpression, a phenomenon that has been reported previously (Paulsen et al. 2009; Salas-Armenteros et al. 2017). The increase in R-loops was effectively recapitulated in cells treated with different siRNAs against the RTEL1 transcripts, an accumulation that was also inhibited by the induction of RNase H (Fig. 4E), indicating that the increase in R-loops upon RTEL1 siRNA treatment is genuine. Likewise, CRISPR/Cas9-based targeted knockout of RTEL1 lead to a marked increase in R-loop accumulation, an effect that was also reversed in cells overexpressing RNase H (Fig. 4C,D; Supplemental Fig. S3E), Notably, elevated R-loops were also detected in RTEL1-depleted normal human diploid cells RPE-1 (Supplemental Fig. S3F), thereby excluding the possibility that the R-loop-antagonizing function of RTEL1 is restricted to cancer cells.

To further rule out the possibility of nonspecific binding of the S9.6 antibody, we tested the enrichment of R-loops after RTEL1 loss using U2OS cells stably expressing HB-GFP, a fusion of green fluorescent protein (GFP) with the DNA–RNA hybrid-binding (HB) domain of RNase H that can be used to detect R-loops in cells (Tanikawa et al. 2016). Indeed, RTEL1 ablation resulted in R-loop accumulation compared with control cells in this setting, as judged by the increased nuclear GFP intensity in RTEL1-depleted cells (Fig. 4F), suggesting that the aberrantly enhanced accumulation of R-loops after RTEL1 loss is genuine. As our evidence suggests that RTEL1 and Poldip3 reside in a physical complex with mutually dependent functions on chromatin, we assessed whether these proteins function epistatically to protect cells against R-loop accumulation. Indeed, U2OS cells codepleted of RTEL1 and Poldip3 showed no additional increase in R-loops compared with cells depleted for either protein alone (Supplemental Fig. S3G), indicating that RTEL1 and Poldip3 function in the same pathway. To further validate the notion that loss of RTEL1 leads to increased R-loop accumulation, we isolated genomic DNA from U2OS cells with and without RTEL1 and performed a dot blot analysis using the S9.6 antibody to probe for R-loops. Again, we found an increase in the amount of S9.6 signal in the genomic DNA of U2OS cells depleted of RTEL1 as compared with siRNA-treated control cells or RTEL1-depleted cells subsequently incubated with RNase H (Fig. 4G,I). Similarly, Poldip3 loss also leads to a marked increase of genomic R-loops as detected by the dot-blot analysis compared with siRNA control cells or in DNA from Poldip3-ablated cells subsequently incubated with RNase H (Fig. 4H,I), further implicating these proteins in preventing excessive accumulation of potentially harmful R-loops.

Next, we asked whether RTEL1 might physically interact with RNA:DNA hybrids. Using the S9.6 antibody affinity purification approach in the presence of excess RNase A (to reduce unspecific RNA-mediated interactions and avoid S9.6 recognition of double-stranded RNA [dsRNA]), we were able to pull down endogenous RTEL1, whereas pretreatment of nuclear extracts with the DNase prevented RTEL1 pull-down, indicating specific binding of RTEL1 to the RNA:DNA hybrid (Fig. 4J). Interestingly, RTEL1 was pulled down stronger after CPT treatment (Fig. 4J), indicating that CPT-induced R-loop accumulation augmented RTEL1 binding to RNA:DNA hybrids. We then assessed whether RTEL1 could bind pure RNA:DNA hybrids in vitro. Using immobilized biotin-labeled RNA and DNA oligonucleotides annealed in vitro, we found that stably expressed wild-type GFP-RTEL1 was efficiently retrieved in biotin pull-downs (Fig. 4K). As a control, an annealed biotin-labeled D-loop was tested for its ability to pull down GFP-RTEL1. We found that RTEL1 was retrieved by both biotin R-loops or D-loops, indicating that RTEL1 binds R-loops as efficiently as D-loops. Treatment of annealed biotin R-loops with pure RNase H prior to pull-down also reduced the coelution of RTEL1 (Fig. 4K), indicating that the binding of RTEL1 to R-loops is genuine.

### Genomic localization of R-loops after RTEL1 and Poldip3 loss

**Figure 5.**
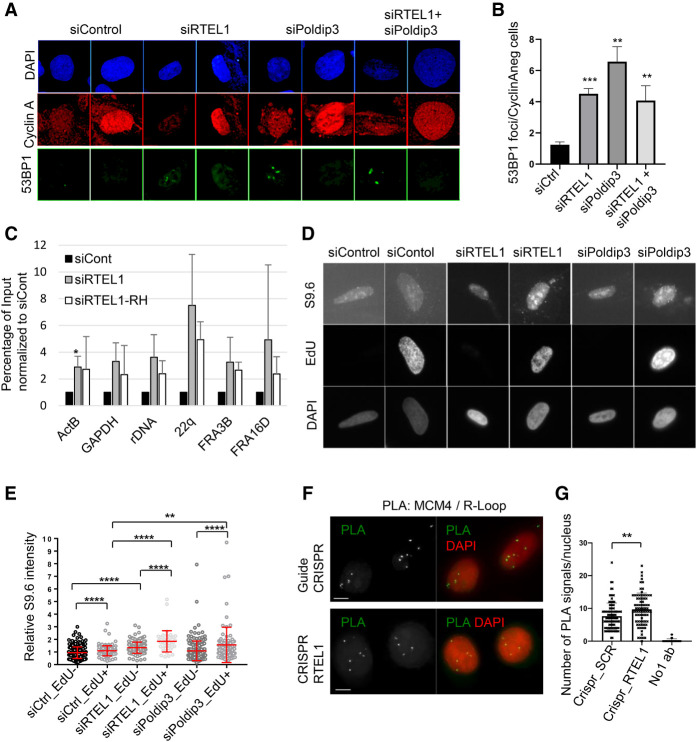
R loops accumulate at specific genomic loci and associate with replication after RTEL1 depletion. (*A*) Increased 53BP1 bodies in Cyclin A-negative U2OS cells depleted of RTEL1, Poldip3, or RTEL1 and Poldip3. Representative images showing DAPI and 53BP1 staining in U2OS cells treated with control, RTEL1, Poldip3, or RTEL1 and Poldip3 siRNA. (*B*) Quantification of *A.* The average of three independent experiments are shown. (**) *P* < 0.01; (***) *P* < 0.001, Student's *t*-test. (*C*) DNA:RNA hybrids accumulate at commonly expressed genes (*GAPDH* and *ActB*), common fragile sites (*FRA3B* and *FRA16D*), telomeres (*22q*) and *rDNA* in U2OS cells depleted of RTEL1. Results are obtained with qPCR from DNA samples captured by DNA–RNA immunoprecipitation (DRIP) from siCont or siRTEL1 U2OS samples untreated or treated with RNase H (RH). Percentage of input normalized to siCont from three experiments are shown. (*) *P* < 0.01, Student's *t*-test. (*D*) Increased amount of R loops in EdU-positive RTEL1- or Poldip3-depleted cells. Representative images of S9.6, EdU, and DAPI staining in RTEL1- and Poldip3-depleted cells. (*E*) Quantification of S9.6 nuclear fluorescence intensity in control, RTEL1 or Poldip3 siRNA-treated EdU-positive or EdU-negative U2OS cells. Values normalized to siCtrl. Mean and SD are plotted (**) *P* < 0.01; (****) *P* < 0.0001, two-tailed Mann-Whitney test. (*F*) Proximity ligation assay (PLA) of S9.6 and MCM4 after RTEL1 knockout. HeLa cells sorted (GFP) 48 h after transfection with either CRISPR–Cas9 against RTEL1 or CRISPR control and put on slides. PLA revealing MCM4 and R-loops. (*G*) Quantification of *F*. (SCR) Scrambled control guide. (**) *P* < 0.01, Mann-Whitney test.

Given that RTEL1 and Poldip3 knockdown resulted in increased R loop levels, we set out to explore in more detail the genomic regions showing abundant R-loops in such cells. Common R-loop-prone loci are typically very long genes vulnerable to replication–transcription complexes collisions (Helmrich et al. 2011). Such affected loci, also known as common fragile sites (CFS), are readily revealed as chromosome breaks after replication inhibition by aphidicolin (Aph) treatment and, if unrepaired before mitosis, such persistent replication stress-associated lesions including CFS are marked as 53BP1 bodies in the following G1 cell cycle phase (Lukas et al. 2011). Indeed, we found that RTEL1 and Poldip3 depletion lead to an increased number of nuclear 53BP1 bodies in G1 of U2OS cells (Fig. 5A,B), suggesting that RTEL1/Poldip3 deficiency and R-loops accumulation are associated with enhanced replication stress and CFS expression. To further understand whether CFSs and other genomic loci intersect with R-loop hotspots after RTEL1 and Poldip3 deficiency, we assessed three genomic regions in more detail using the DNA–RNA immunoprecipitation (DRIP) assay. We focused on CFSs (represented by *FRA3B* and *FRA16D*) and other likely R-loop-prone loci in the genome; namely, telomeric repeats (represented by *22q*), repetitive ribosomal DNA (*rDNA*) regions, and highly transcribed genes (*GAPDH* and *ActB*). Indeed, we found that both RTEL1 and Poldip3 depletion increased the R-loop accumulation at all the above-mentioned genomic regions, but not at a control region (Rag1), as judged by the increased pull-down of these DNA regions with the S9.6 antibody (Fig. 5C; Supplemental Fig. S4A,B). Furthermore, overexpression of RNase H reduced the DRIP assay values of these loci indicating that the R-loop accumulation at these genomic sites is genuine. Finally, considering that R-loop accumulation has detrimental consequences, particularly during transcription and replication, we tested whether RTEL1 knockdown has any effect on these processes. RTEL1 siRNA-treated cells showed normal staining with the modified nucleotide ethynyl uridine (EU) (a marker for RNA synthesis) (Supplemental Fig. S4C,D), suggesting that global transcription was unaltered in RTEL1-depleted cells. In contrast, incorporation of 5-ethynyl-2′-deoxyuridine (Edu; a marker for DNA synthesis) (Supplemental Fig. S4E,F) was reduced in siRTEL1 cells, which is consistent with previous publications (Uringa et al. 2012; Vannier et al. 2013) supporting a role for RTEL1 in replication. We therefore asked whether the R-loop accumulation after RTEL1 or Poldip3 depletion is confined to actively replicating cells in S phase by comparing R-loops in cells with the neighboring cells outside S phase. Here we found an enrichment of R-loops in S-phase cells (Fig. 5E), indicating that R-loop accumulation after RTEL1/Poldip3 loss occurs mainly during DNA replication. An increase in R-loops was also detected in non-S-phase cells, although to a lesser degree compared with S phase, suggesting that RTEL1 and Poldip3 are needed for R-loop prevention/resolution throughout the cell cycle. We further examined whether R-loop accumulation after RTEL1 loss might occur in close proximity to replication forks. Indeed, using the proximity ligation assay (PLA), we found that R-loops are enriched in proximity to the replisome helicase component MCM4 (Fig. 5F,G), suggesting that RTEL1 ablation leads to R-loop accumulation preferentially at replication forks.

Collectively, these data suggest that RTEL1 and Poldip3 prevent potential R-loop accumulation, a so-far unsuspected role that helps protect genome integrity at vulnerable regions such as CFS or rDNA and is particularly apparent during DNA replication.

## Discussion

**Figure 6.**
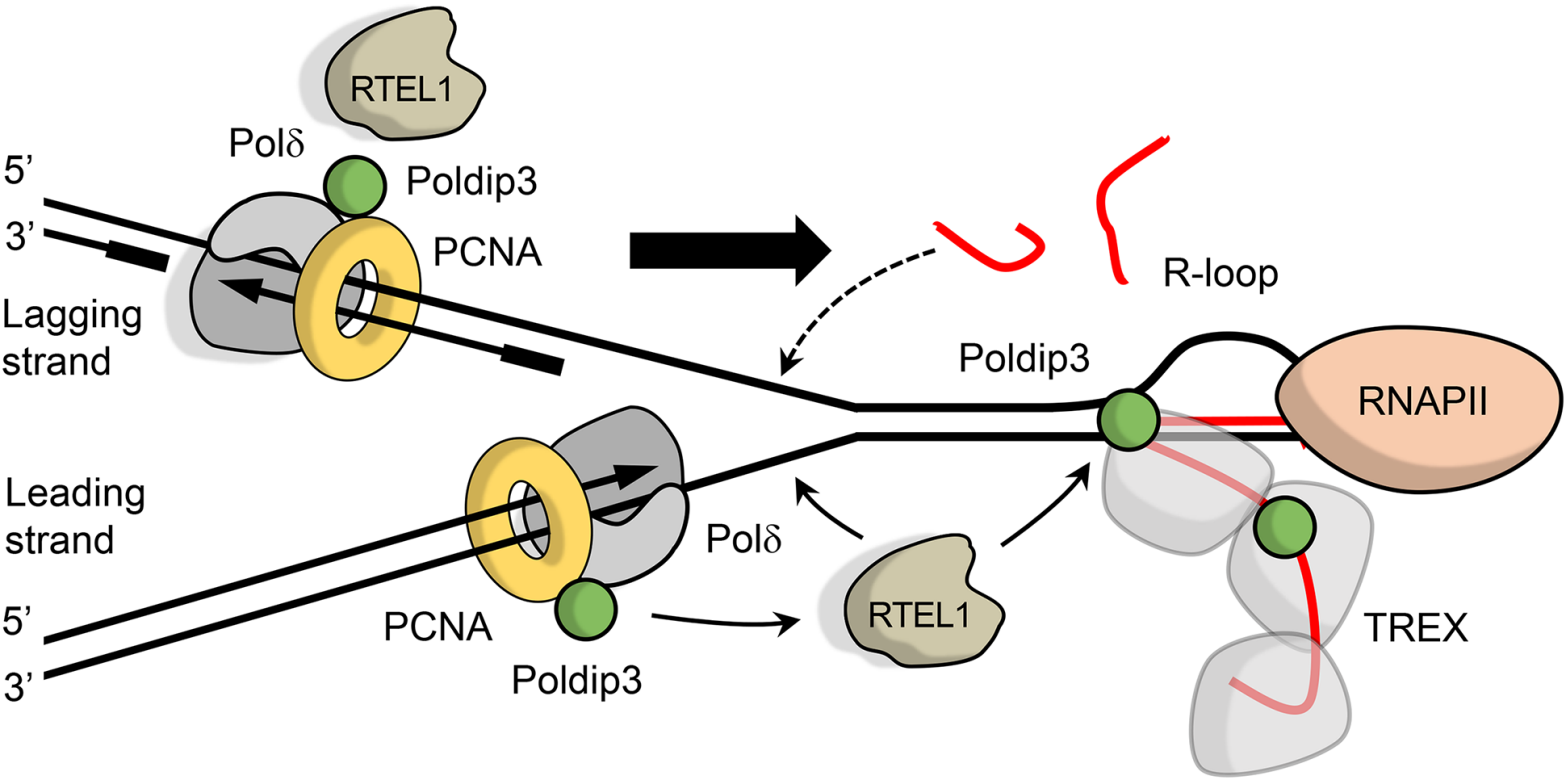
Cartoon showing our model of the role of RTEL1 and Poldip3 in R-loop resolution. RTEL1 associates with the replisome, here represented by Polδ (gray) and PCNA (yellow) via Poldip3 and/or PCNA, as it replicates DNA. As the replication fork encounters the transcription machinery, RTEL1 might resolve R-loops that block the replication fork and/or the R-loops provoked by stalling of replication (shown by arrows from RTEL1). (RNAPII) RNA polymerase II. The black lines represent single-stranded DNA and the red lines depict RNA. The big arrow indicates the direction of the replication fork.

Overall, our present study identifies a previously unrecognized protein complex of RTEL1 and Poldip3 that operates at the crossroad of the fundamental cellular processes of DNA replication and transcription, as schematically depicted in our model presented in Figure 6. Excessive accumulation of R-loops represents a formidable barrier to the replication fork progression and needs to be reversed to prevent replication failure and genomic instability (Heath et al. 2016). The surprising R-loop accumulation when RTEL1 is lost might have two likely causes that in both cases might be explained by a defective physical interaction between RTEL1 and Poldip3. Poldip3 functions as a subunit in both the DNA polymerase δ (Polδ) complex during replication and in the RNA sequestering TREX complex during transcription.

Our observation that RTEL1 and Poldip3 reside in a complex thus indicates that RTEL1 functions in either replication, as previously proposed (Vannier et al. 2013), and/or transcription. RTEL1 loss could lead to defective replication fork progression and replication fork collapse that would result in naked DNA strands accessible to RNA species consequently producing excessive DNA:RNA hybrids. Alternatively, defective TREX complex function could result in untimely RNA sequestration of newly transcribed RNA and an increased pool of RNA moieties competent to invade adjoining DNA template strands. Our results that RTEL1 and Poldip3 loss lead to R-loop accumulation most prominently in S phase during DNA replication and in a close proximity to the replisome (Fig. 5D–G) and RTEL1 depletion has no overt effect on global transcription (Supplemental Fig. S4C,D) points mainly to the first possibility. We hypothesize that Poldip3 as a subunit of Polδ together with other components of the replisome (e.g., PCNA) are critical for the timely recruitment of RTEL1 to the stalled replication fork where it resolves replication fork stalling at sites of transcription. One likely obstacle to replication forks is the RNA polymerase as well as other components of the transcription machinery. This is also supported by the increased R-loop accumulation at CFSs in RTEL1- or Poldip3-depleted cells (Fig. 5C; Supplemental Fig. S4A,B), which are located in very long genes that often replicate late in S phase and are thus more sensitive to replication stress-inducing replication–transcription conflicts. We propose that RTEL1 might strip off such transcription proteins from DNA before they collide with the replisome. As such, the function of RTEL1 would resemble that of its ancient ortholog in bacteria, DinG, which functions to prevent collisions between replication forks and the RNA polymerase (Boubakri et al. 2010). This notion is consistent with the previous reports demonstrating that RTEL1-deficient cells are hypersensitive specifically to topoisomerase I (TOPOI) poisons (Barber et al. 2008), agents that induce increase of R-loops specifically at highly transcribed regions (Marinello et al. 2013). Likewise, POLδ deficiency also renders cells hypersensitive to TOPOI inhibitors (Tumini et al. 2016), raising the possibility that both RTEL1 and POLδ play a critical role in overcoming TOPOI-induced DNA damage during replication. In further support of this concept, two reports published at the time of submission of our revised manuscript suggest that RTEL1 is involved in suppressing replication–transcription conflicts (Takedachi et al. 2020; Wu et al. 2020) and R-loop accumulation (Wu et al. 2020).

Last but not least, this previously unsuspected role of the human RTEL1–Poldip3 complex, identified in our present study, contributes to genome integrity maintenance under replication stress, an important homeostatic function with implications for proper organismal development and avoidance of a range of grave pathologies including cancer.

## Materials and methods

### Mass spectrometry analysis

SDS-PAGE-resolved, 4%–12% gradient gels were silver-stained with SilverQuest (Life Technologies), and appropriate bands were cut and subjected to in-gel digestion followed by liquid chromatography-MS with the Q Exactive HF mass spectrometer (Thermo Fisher Scientific). Resulting peptides were identified by protein-sequence database searches using MaxQuant software (Tyanova et al. 2016).

### Plasmids and gene silencing

A cDNA for human RTEL1, KIAA1088, was obtained from HUGE protein database (http://www.kazusa.or.jp/techcgi/view_direct.cgi?id=hk02589s1) and was inserted by PCR into pFlag-CMV2 (Sigma) or into the pEGFP-C1 vector (Clontech). To generate GFP-RTEL1 mutant versions, the causative dyskeratosis congenita mutations were introduced by standard PCR using mutant primers and the same flanking primers as used to generate GFP-RTEL1 WT (Schmid et al. 2018).

All plasmid transfections were performed using FuGene6 (Roche). siRNA oligonucleotides (Dharmacon) were synthesized to the following human sequences: (sense strand) siRTEL1#1 (5′-UGAAGAAACAAAGAGUAAUU-3′) and (antisense strand) siRTEL1#1 (3′-UUACUUCUUUGUUCUCUCAUU-5′), (sense strand) siRTEL1#2 (5′-GCCUGUGUGUGGAGUAUGA-3′) and (antisense strand) siRTEL1#2 (5′-UCAUACUCCACACACAGGC-3′), and (sense strand) siPOLDIP3 (5′-GGGAAAGUGCAGGAUGCCA-3′) and (antisense strand) (5′-UGGCAUCCUGCACUUUCCC-3′). For efficient knockdown of RTEL1 and Poldip3 in all experiments, cells were transfected twice with the siRNAs as follows. On day 1, cells were incubated for 6 h with transfection reagents containing control or target siRNA. Then the transfection medium was replaced with fresh growth medium and cells were incubated for an additional 18 h. On day 2, cells were treated as in day 1 and then incubated for an additional 42 h. siCONTROL (Dharmacon) was used as a control siRNA. All siRNA transfections were performed with 100 nM siRNA duplexes using Lipofectamine RNAi MAX (Invitrogen). Cell culture human U2OS and HEK293T were cultured in DMEM containing 10% fetal bovine serum. U2OS-derived cell lines capable of expressing ectopic RTEL1 alleles from pEGFP-C1-RTEL1 constructs were generated and maintained as described.

Additional information about the Materials and Methods is available in the Supplemental Material.

## Supplementary Material

Supplemental Material
