# Supplementary material for: Human RTEL1 associates with Poldip3 to facilitate responses to replication stress and R-loop resolution
Source: Genes Dev. 2020 Aug 1;34(15-16):1065–74. doi: 10.1101/gad.330050.119 (PMC7397856; doi:10.1101/gad.330050.119)
Supplement: Supplemental Material [file supp_34_15-16_1065__index.html]

Supplemental Material 

# Human RTEL1 associates with Poldip3 to facilitate responses to replication stress and R-loop resolution

## Supplemental Material

- Supplemental\_Material.pdf
