# Supplementary material for: Human RTEL1 associates with Poldip3 to facilitate responses to replication stress and R-loop resolution
Source: Genes Dev. 2020 Aug 1;34(15-16):1065–74. doi: 10.1101/gad.330050.119 (PMC7397856; doi:10.1101/gad.330050.119)
Supplement: Supplemental Material [file supp_gad.330050.119_Supplemental_Material_.pdf]

Supplemental Information

Björkman Suppl. Fig S1

A

	CHFR#1	**RTEL1 #1**	CHFR#2	**RTEL1#2**
RTEL1	4	**52**	9	**65**
ILF3	22	**39**	4	**39**
DDX54	4	**27**	1	**28**
FXR2	1	**26**	1	**27**
HIST1H1B	4	**24**	15	**25**
HIST1H1E;HIST1H1D	9	**24**	21	**24**
FMR1	1	**23**	1	**25**
TUBB4A	14	**22**	19	**19**
TUBA1C;TUBA1B	17	**22**	20	**22**
USP36	1	**21**	1	**25**
TUBB2A;TUBB2B	16	**20**	20	**20**
ADAR	1	**20**	1	**19**
HSPA2	13	**19**	17	**22**
CCDC86	1	**19**	4	**21**
HNRNPA1;HNRNPA1L2	12	**18**	10	**19**
OASL	1	**17**	5	**16**
POLDIP3	1	**17**	6	**9**
ALYREF	4	**17**	7	**17**
UTP14A	1	**16**	1	**16**
MOV10	1	**16**	1	**18**
WDR36	1	**15**	1	**17**
HSPA8	11	**15**	14	**14**
RRP1B	1	**15**	1	**14**
H2AFY	1	**14**	1	**15**
NUSAP1	1	**14**	1	**13**
Actin	12	**13**	12	**11**
DAP3	1	**12**	2	**14**
DDX50	2	**12**	2	**9**
SRP68	1	**12**	1	**12**
TOP2B	1	**12**	1	**15**
SAFB2	1	**12**	1	**16**
NUFIP2	1	**11**	7	**10**
MRPS22	1	**10**	1	**9**
TBL3	1	**10**	1	**10**
C12orf43	1	**10**	1	**10**
C16orf88	1	**10**	1	**10**
C17orf85	1	**10**	1	**10**
NOP16	1	**10**	1	**9**
STRBP	3	**10**	1	**11**
RBM19	1	**10**	1	**11**
GLYR1	1	**10**	1	**11**
C7orf50	1	**9**	3	**9**
SURF6	1	**9**	1	**10**
ZFR	1	**9**	3	**9**
YTHDC2	1	**9**	1	**10**
MKI67IP	1	**8**	1	**9**
GPATCH4	1	**8**	1	**9**
MMS19	1	**1**	1	**11**

B

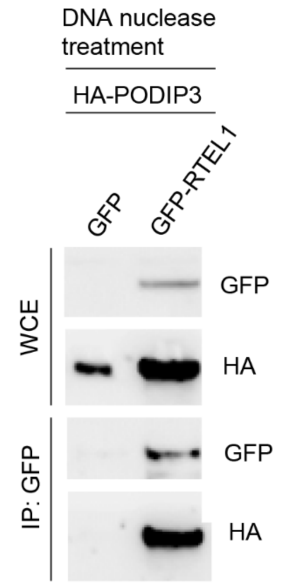

Supplementary figure 1

(*A*) RTEL1 interactors identified by mass spectrometry analysis. The table shows the interactors with the highest peptide intensity values estimated from the ratios between the FLAG-RTEL1 and the FLAG-CHFR proteins.

(*B*) GFP-RTEL1 binds HA-Poldip3 after Dnase treatment (Benzonase) of whole cell extracts. HEK293T cells were co-transfected with HA-Poldip3 and either GFP or GFP-RTEL1 and incubated for 24h prior to harvest. Whole cell extracts were treated with micrococcal nuclease and DNase I for 30 min at 37°C temperature prior to GFP immunoprecipitation followed by immunoblotting with HA and GFP antibodies.

Björkman Suppl. Fig S2

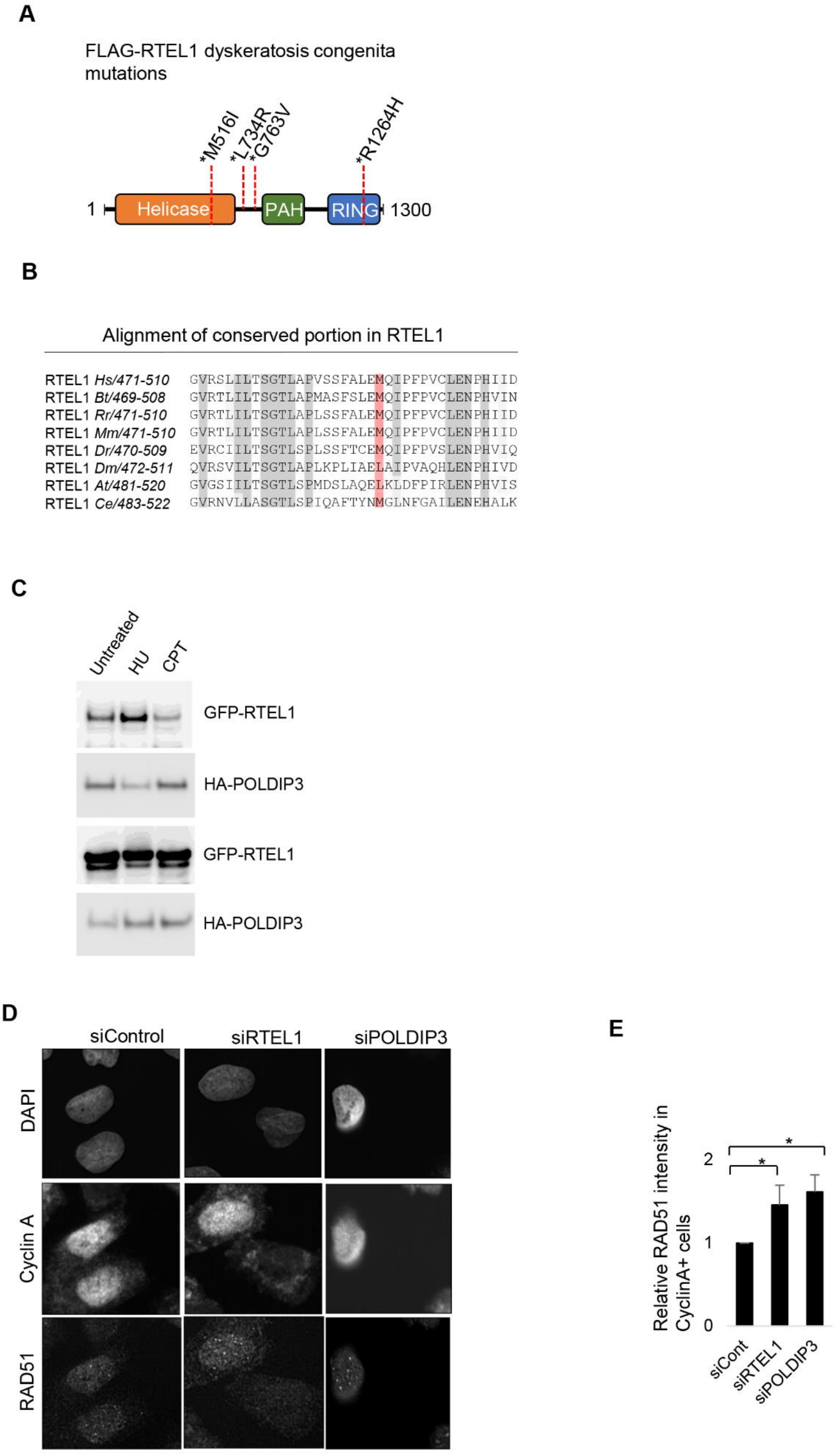

Supplemental figure 2

(*A*) Model showing a panel of RTEL1 mutations identified in dyskeratosis congenita/Hoyeral-Hreiderson syndrome patients.

(*B*) Alignment of homologous amino acid sequences flanking the conserved M516I mutation in RTEL1. The conserved methionine is highlighted in red.

(*C*) Immunoprecipitation of GFP-RTEL1 after replication stress. U2OS cells stably expressing GFP-RTEL1 were transfected with HA-tagged Poldip3 and incubated for an additional 24 hours. Subsequently cells were either left untreated or treated with HU (2 mM) or CPT (100 μM) for an additional 24 hours. Cells were harvested and lysates subjected to GFP pulldown. Resolved protein complexes were probed with the indicated antibodies.

(*D*) Representative images of DAPI, RAD51 and Cyclin A immunofluorescence staining in U2OS cells treated with control, RTEL1 or POLDIP3 siRNA.

(*E*) Quantification of (*A*). Average RAD51 immunofluorescence intensity in Cyclin A positive cells. Student's t-test *$p<0.05$

Björkman Suppl. Fig S3

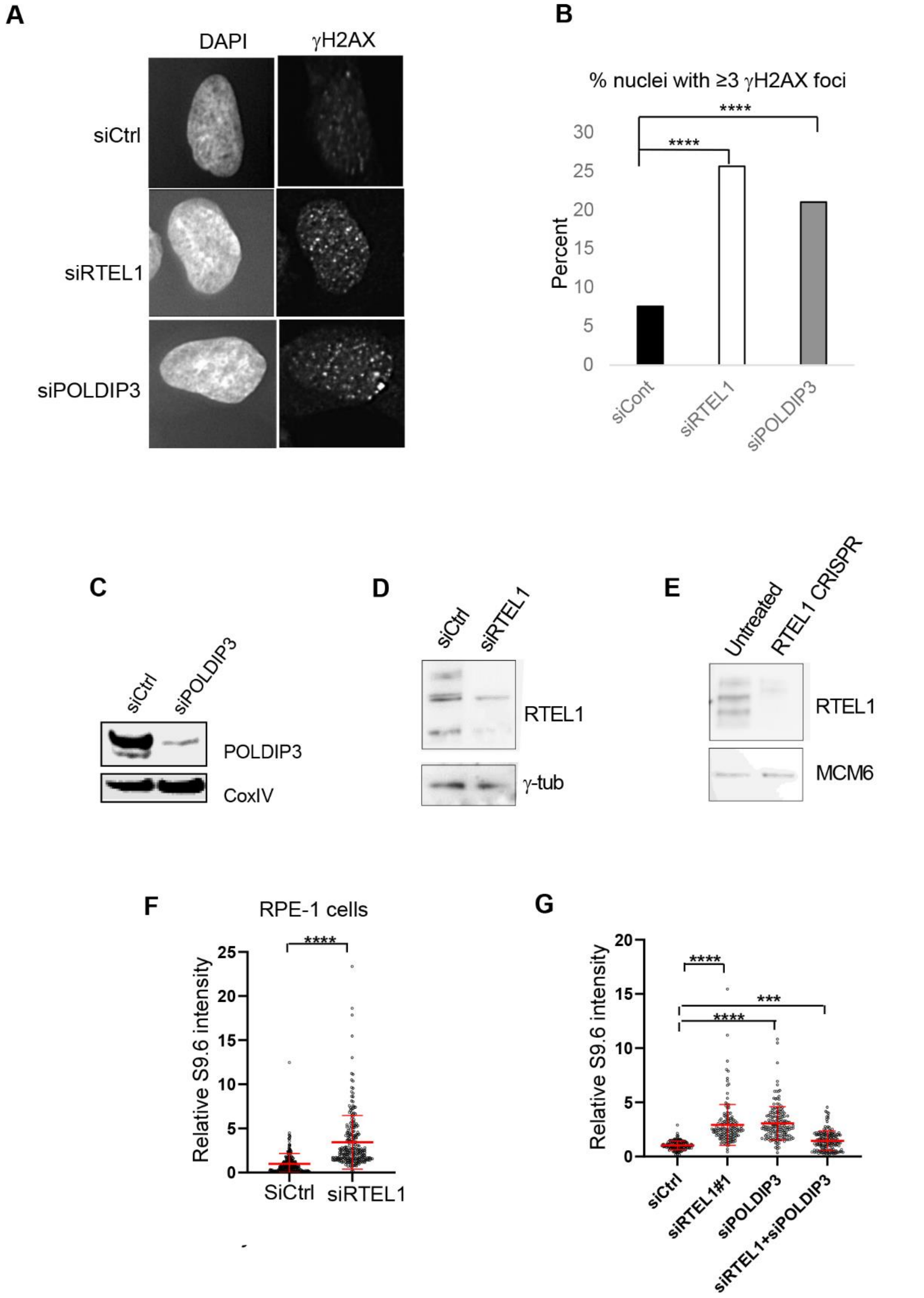

Supplemental figure 3

(A) Representative images of γH2AX fluorescence and DAPI staining in U2OS cells treated with control, RTEL1 or Poldip3 siRNA.

(*B*) Quantification of γH2AX foci. Percentage of nuclei with ≥3 γH2AX foci in U2OS cells treated with control, RTEL1 or Poldip3 siRNA are shown. Chi-square test. ****$p<0.0001$.

(*C*) Knockdown of Poldip3 in U2OS cells using Poldip3 siRNA. Cells were transfected two times (each transfection separated be 24 hours) with either control or Poldip3 siRNA and incubated for an additional 48 hours. Cells were harvested, proteins resolved by SDS-PAGE and proteins probed by the indicated antibodies.

(*D*) Knockdown of RTEL1 in U2OS cells using RTEL1 siRNA. Cells were transfected two times (each transfection separated be 24 hours) with either control or RTEL1 siRNA and incubated for an additional 48 hours. Cells were harvested in CSK buffer, proteins resolved by SDS-PAGE and proteins probed by the indicated antibodies.

(*E*) Knockout of RTEL1 using RTEL1 CRISPR/CAS9. HEK293T cells were left untreated (control) or transfected two times (each transfection separated be 24 hours) with RTEL1 CRISPR and incubated for an additional 48 hours. Cells were harvested in CSK buffer, proteins resolved by SDS-PAGE and proteins probed by the indicated antibodies.

(*F*) Increased accumulation of R-loops in RPE-1 cells depleted of RTEL1. Quantification of nuclear S9.6 fluorescence intensity in RPE-1 cells treated with control or RTEL1 siRNA.

(*G*) Co-depletion of RTEL1 and Poldip3. U2OS cells were transfected with either RTEL1 siRNA, Poldip3 siRNA or both RTEL1 and Poldip3, and incubated for an additional three days. R-loops were probed with the S9.6 antibody and cells were analysed by fluorescence microscopy.

Values normalized to siCtrl. Mean and SD are plotted *** $p<0.001$, ****$p<0.0001$, two-tailed Mann-Whitney test (D and E)

Björkman Suppl. Fig S4

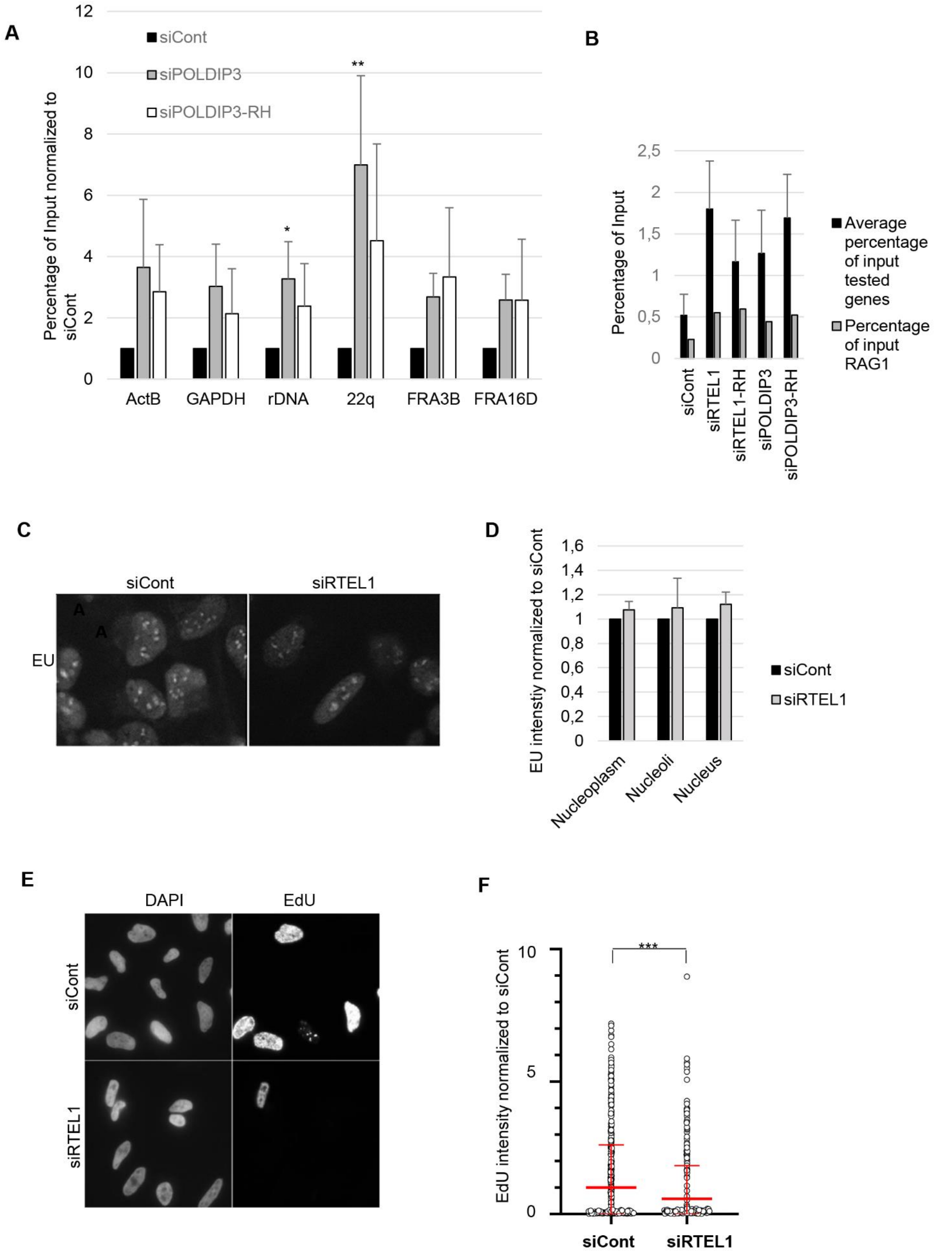

Supplemental figure 4

(A) DRIP assay of cells depleted for Poldip3. Percentage of input normalized to siCont are shown. Student's t-test $**p<0.01$, $*p<0.05$

(B) DRIP assay of cells depleted for RTEL1 or Poldip3. Percentage of input of *RAG1* (non-expressed, negative control) gene in comparison to average percentage of input of *ActB*, *GADPH*, *rDNA*, *22q*, *FRA3B* and *FRA16D*.

(C) Representative images of EU immunofluorescence staining in U2OS cells treated with control or RTEL1 siRNA.

(D) Quantification of *(A)* in nucleus, nucleoli and nucleoplasm.

(E) Representative images of DAPI and EdU immunofluorescence staining in U2OS cells treated with control or RTEL1 siRNA.

(F) Quantification of *(C)* Student's t-test. $***p<0.001$

Materials and methods

Cell culture

Human U2OS, RPE-1 and HEK293T were cultured in DMEM containing 10% fetal bovine serum. U2OS derived cell lines capable of expressing ectopic RTEL1 alleles from pEGFP-N1-RTEL1 constructs were generated and maintained as described. U2OS cells stably expressing inducible RNaseH-GFP construct were untreated or treated with 1μg/ml Doxocyclin for 48H before R loop immunofluorescence staining. U2OS cells transfected with RTEL1 crispr-cas9 or control guide crispr-cas9 plasmids 2 days prior to FACS sorting and let to grow on coverslips for 5 days before immunofluorescence staining. On day 3 cells were transfected with RNaseH-GFP or GFP plasmids.

Plasmids and gene silencing

A cDNA for human RTEL1, KIAA1088, was obtained from HUGE protein database (www.kazusa.or.jp/techcgi/view_direct.cgi?id=hk02589s1) and was inserted by PCR into pFLAG-CMV2 (Sigma) or into the pEGFP-C1 vector (Clontech). The GFP-RTEL1 mutant constructs were introduced using the QuikChange Site Directed Mutagenesis Kit (Stratagene) or by a two-step PCR protocol as described previously (Schmid et al., 2018). All plasmid transfections were performed using FuGene6 (Roche). RNAseH1 was PCR-amplified from pEGFP-C2-RNAseH1 using AccuPrime™ Pfx DNA Polymerase (Invitrogen, # 12344-024) and the following primers (F: 5'-GTCGAC AGCTGGCTTCTGTTCCTG, R: 5'- GCGGCCGC TCAGTCTTCCGATTGTTTAGC). The PCR product was then inserted into SalI and NotI-digested pENTR and subsequently cloned into a modified (DOX inducible and GFP containing) pInducer20 plasmid using the Gateway cloning

system. Viral particles were produced by transfecting the lentiviral -and corresponding packaging plasmids (encoding polymerase and envelope proteins) into HEK293T cells. Supernatants were collected two times with 24 hours intervals, filtered and used to infect U2OS. The cells (DOX-sheIF4A3-U2OS) were selected in the presence of 10 μg/mL blasticidin for 72 hours or until all non-transduced control cells were killed. siRNA oligonucleotides (Dharmacon) were synthesized to the following human sequences (sense strand): siRTEL1#1 (5'- UGAAGAAACAAAGAGUAAUU -3') and (antisense strand) siRTEL1#1 (3' – UUACUUCUUUGUUCUCUCAUU – 5'), (sense strand) siRTEL1#2 (5' – GCCUGUGUGUGGAGUAUGA -3') and (antisense strand) siRTEL1#2 (5' – UCAUACUCCACACACAGGC -3'), (sense strand) siPOLDIP3 (5' – GGGAAAGUGCAGGAUGCCA – 3') and (antisense strand) (5' – UGGCAUCCUGCACUUUCCC -3'). For efficient knockdown of RTEL1 and POLDIP3 in all experiments, cells were transfected twice with the siRNAs as follows. On day one, cells were incubated for 6 hours with transfection reagents containing control or target siRNA. Then the transfection medium was replaced with fresh growth medium and cells were incubated for an additional 18 hours. On day two, cells were treated as in day one and then incubated for additional 42 hours. siCONTROL (Dharmacon) was used as a control siRNA. All siRNA transfections were performed with 100 nM siRNA duplexes using Lipofectamine RNAi MAX (Invitrogen).

Immunochemical methods and purification of recombinant proteins

Immunoblotting, immunoprecipitation, and immunofluorescence were done as described elsewhere. Antibodies used in this study included rabbit polyclonals Poldip3 (Bethyl), POLD3 (Abcam) and mouse POLD1 (BD Biosciences). R-loop antibody used was S9.6 (MABE1095, EMD Merck). Antibodies to FLAG, MYC, HA, GFP, actin and MCM6 were described previously. Rabbit RTEL1

antibody was generated as described previously (Schertzer et al. 2015). To isolate HA-tagged proteins, a high-salt purification protocol was used as previously described (Unsal-Kacmaz and Sancar 2004). Briefly, HEK293T whole cell extracts were incubated with anti-HA conjugated protein A beads (Sigma) at 4°C for two hours followed by three washes with lysis buffer (50 mM Tris-HCl pH 7.5, 140 mM NaCl, 0.5% NP-40, protease inhibitor cocktail (Roche)). Bound HA-tagged proteins were purified by subjecting the beads to three washes with a high-salt buffer (750 mM NaCl, 50 mM Tris-HCl pH 7.5 and protease inhibitor cocktail). The resin was then washed two times in a low-salt buffer ((140 mM NaCl, 50 mM Tris-HCl pH 7.5) and HA-tagged proteins were eluted with 4x sample buffer.

For γH2AX, 53BP1, RAD51 and CyclinA immunofluorescence staining, cells were let to grow on 96-well imaging plates. In brief, cells were washed 2X in phosphate-buffered saline (PBS), incubated with 4% paraformaldehyde for 7 min. The cells were then washed 1X in PBS and incubated with ice-cold methanol for 2 min. Subsequently, the cells were washed 1X in PBS and blocked for 30 min in blocking buffer (Tris-buffered saline, 0.05%Tween20 (TBST), 2% bovine serum albumin) and incubated with γH2AX (Millipore, 05-636, 1:500) antibody or Cyclin A2 antibody (Santacruz, sc-271682, 1:300) and RAD51 (Santacruz, sc-8349, 1:500) or 53BP1 (NB100-304, 1:500) and Cyclin A2 (Proteintech 66391-1-Ig, 1:500) in blocking buffer at 4°C overnight. The following day, the cells were washed 3X times in TBST and incubated with secondary antibodies Alexa-Fluor (1:800) and DAPI (0.5μg/ml) in blocking buffer for 1H at RT. The cells were then washed 1X in TBST, followed by 3X in PBS. Images were acquired on InCell Analyzer 2200 fluorescence microscope using 40x objective and fluorescence intensities or foci were calculated with Cellprofiler.

EU staining

For 5-Ethynyl Uridine (EU) staining, cells were let to grow on 96-well imaging plates and incubated with 200μM EU for 30 min. Thereafter cells were fixed in ice-cold methanol for 5 min followed by permeabilization with 0.5% Triton-X for 10 min. The cells were then washed 3X with PBS, incubated with EU click-it mixture (Click-iT™ RNA Alexa Fluor™ 488 Imaging Kit, Invitrogen) for 30 min, rinsed with component F, followed by 3X washes with PBS, incubation with DAPI for 1H and 3X washes with PBS. Images were acquired on InCell Analyzer 2200 fluorescence microscope using 40x objective and fluorescence intensities or foci were calculated with Cellprofiler.

R loop immunofluorescence staining

For R loop detection by immunofluorescence, cells were grow on coverslips and pre-extracted with CSK buffer (10nM PIPES pH.6.8, 100mM NaCl, 300mM sucrose, 3mM MgCl2, 1mM EGTA pH5.7, 0.5% TX100) for 5min on ice, washed 5X with cold PBS, before fixation in mSTF buffer (150mM Bronopol, 108mM diazolidinyl urea, 10mM NaCitrate, 50mM EDTA) for 30min. The cells were subsequently washed 2X with cold PBS and permeabilized in PB buffer (100mM Tris-HCl pH 7.4, 50mM EDTA pH 8.0, 0.5% TX100) for 15min, washed 2X in PBS, blocked for 30min in blocking buffer (Tris-buffered saline, 0.05%Tween20, 2% bovine serum albumin) and incubated with S9.6 (MABE1095, EMD Merck, 1:500) or GFP (sc-9996, SantaCruz, 1:500) antibody at 4°C overnight, thereafter washed 3X with PBS, incubated with secondary antibodies Alexa-Fluor (1:800) for 1H at room temperature, washed 3X with PBS and mounted on glass slides with ProLong™ Gold Antifade Mountant with DAPI (Thermofisher). Cells co-stained with EdU were incubated with EdU (10μM) for 30 min prior to pre-extraction and incubated with Edu Click-it cocktail (100mM Ascorbic Acid,

6mM $CuSO_4$, 4μM Alexa Fluor 647 azide in PBS) for 30 min and washed 2X in PBS before incubation with secondary antibody. Images were acquired on NikonTi2 fluorescence microscope using 60x objective. Fluorescence intensities were calculated with Cellprofiler.

qPCR

DNA recovered from DRIP assay were quantified by qPCR in QuantStudio™ 5 System. Samples were run in triplicates using Power SYBR Green PCR master mix according to manufacturer's instructions. The comparative CT method was used. For RAG1 primers Forward: 5'-CGGCCGAGTTCGGAGAG-3' and Reverse: 5'-GCGGTGCAACGAATTCCC-3', ActB Forward: 5'- CTTTTATGGCTCGAGCGGC -3' and Reverse: 5'- GGGCTTACCTGGCGGC -3', and GAPDH Forward: 5'- GCGCCCCCGGTTTCTAT-3' and Reverse: 5'-CTCTCCGCCCGTCTTCAC-3' were used. Previously published primers for FRA3B, FRA16D (Wang et al. 2018), rDNA (Urban et al. 2016) and 22q (Sagie et al. 2016) were used.

Cell fractionation

The preparation of chromatin fraction was performed according to the previously described acid extraction protocol (Gong et al. 2010). Briefly, cells were harvested at indicated times either after treatment with camptothecin (100 M). Cells were subsequently lysed with low salt EBC buffer (20 mM Tris-HCl, pH 8.0, 10 mM NaCl, 1.5mM MgCl2, 1 mM EDTA, 0.5% Nonidet P-40 supplemented with protease inhibitors, and chromatin enriched pellets were washed with PBS three times. The insoluble chromatin fractions were resuspended in 0.2 M HCl for 30 min on ice. The resultant soluble extraction was neutralized with 1M Tris-HCl pH 8.5 for further analysis.

In vitro binding assay

HEK293T cells were transfected with HA-Poldip3 and incubated 48 hours prior to cell lysis. HA-Poldip3 was HA immunoprecipitated and immunocomplexes were washed three times in a high ionic strength buffer (500 mM NaCl) to remove associated proteins. Purified GST-tagged RTEL1 proteins were incubated with HA-Poldip3 conjugated beads and subsequently the beads were washed in EBC buffer (140 mM NaCl, 10 mM Tris-HCl, 5 mM EDTA, 0.5% NP-40 and protease inhibitor cocktail (Roche)) and subsequent standard GFP IP. Immunocomplexes were eluted in glycine-HCl buffer proteins probed by western blotting.

Size exclusion chromatography

The HEK293T whole cell extract, prepared by lysing cells in a detergent free buffer (140 mM NaCl, 50 mM Tris-Cl (pH 8.0) 0.2 mM sodium orthovanadate and protease inhibitors) followed by ultrasonication, was loaded on an NaPO4-buffered Superose 6 column 10/300 GL (GE Healthcare Life Sciences), and collected fractions were concentrated by vacuum centrifugation and an equal volume from each fraction was loaded onto a 4-12% gradient SDS–polyacrylamide gel.

Dot blot analysis

Genomic DNA was isolated from control and RTEL1 or Poldip3 RNAi-treated cells using the Dneasy Blood and Tissue Kit (Qiagen) following the manufacturer's instructions. A nylon membrane was hydrated/equilibrated in methanol for 10 minutes, washed two times in TBST buffer and 20 μg of

DNA was hand spotted on the hydrated nylon membrane. Prior to blotting bulk RNA was digested with 100 µg/ml RNase A (Thermo Fisher Scientific). A DNA/RNA sample digested with 2U of RnaseH (Ribonuclease H, Thermofischer Scientific) for 30 min at room temperature was spotted on the membrane as a control. Absorbed DNA/RNA on the still hydrated membrane was UV crosslinked for 10-15 minutes with the membrane face down on a transilluminator equipped with 312nm bulbs. Membranes were blocked in milk buffer and probed with S9.6 antibody over night at C. Total DNA/RNA spotted on the membranes were shown by staining membranes with methylene blue for 5 min followed by multiple washes with PBS. Intensities were quantified in ImageJ.

R-loop binding assays

R-loops and D-loops were generated in vitro as previously described with modifications (Chang et al. 2017). Briefly, R-loop and D-loop hybrids were generated by annealing purified biotinylated DNA 5′-GGGTGAACCTGCAGGTGGGCGGCTGCTCATCGTAGGTTA GTTGGTAGAATTCGGCAGCGTC-3 or non-biotinylated (negative control) DNA 5′-GGGTGAACCTGCAGGTGGGCGGCTGCTCATCGTAGGTTA each with either RNA, 5′-AAAGAUGUCCUAGCAAGGCAC-3′, or DNA, 5′-AAAGATGTCCTAGCAAGGCAC-3′. For immobilization on beads, 10 pmol each of biotinylated or non-biotinylated nucleotide hybrids was incubated with streptavidin-coated magnetic beads (DynaBeads M-280 streptavidin) in modified lysis buffer (50 mM Tris, pH 7.5; 150 mM NaCl; 0.5% NP40; 1 mM DTT) 30 min at room temperature and subsequently washed three times in lysis buffer buffer. For analysis of GFP-RTEL1 binding to nucleotide-conjugated beads, U2OS cells stably expressing GFP-RTEL1 were lysed in lysis buffer (containing protease inhibitor cocktail) and extracts were cleared by sonication and centrifugation at 14,000 rpm. Whole extracts digested with 2U of RnaseH (Ribonuclease H, Thermofischer Scientific)

for 30 min at room temperature was used as a control. Extracts were preincubated with unconjugated streptavidin beads at 4°C for 1 h and incubated with R-loop or D-loop-conjugated beads for 30 min at room temperature. Subsequently beads were washed five times in lysis buffer Proteins were eluted by boiling beads in Laemmli sample buffer for 10 min and analysed by immunoblotting.

DNA-RNA immunoprecipitation (DRIP) assay

The DRIP assay was performed using the anti-DNA-RNA S9.6 antibody as previously described with few modifications. In brief, U2OS cells were treated with either RTEL1, Poldip3, or control siRNAs for three days. Cells were fixed directly in plates with 1% paraformaldehyde (PFA) in PBS for 10 min at room temperature. Fixed cells were scraped off, collected and washed in PBS prior to PFA quenching with glycine (125 mM) for 5 min at room temperature. Pelleted cells were washed twice in PBS and suspended in lysis buffer (50 mM Hepes KOH pH 7.5, 140 mM NaCl, 1 mM EDTA 10% glycerol, 0.5% NP-40, 0.25% Triton X-100) supplemented with Protease inhibitor cocktail (Roche) and incubated for 15 min on ice. Lysed cells were centrifuged at 3000 rpm for 5 min at 4C and pellets were washed twice in lysis buffer (without EDTA) and finally resuspended in 200 ul resuspension buffer (10 mM Tris-HCl pH 8, 100 mM NaCl, 0.1% Na-Deoxycholate, 0.5% N-laurolsarcodine). 200 ul resuspended pellets of each lysate were sonicated for 30 min (30 sec on/ 30 sec off) in a Bioruptor water bath-sonicator followed by centrifugation at 14.000 rpm for 10 min at 4C. Supernatant was used for subsequent immunoprecipitation. DNA concentrations were measured volumes of chromatin were adjusted to 500 ul in resuspension buffer for each immunoprecipitation. Sonicated chromatin was mixed with 30 ul Dynabeads™ Protein G per sample and cleared for 1h at 4C. Immunoprecipitations were performed by mixing cleared-sonicated chromatin with 2 ug/500 ul anti-R-loop (S9.6, MABE1095, EMD Merck) together with 20 ul ul Dynabeads™ Protein G and

incubated at 4C overnight. Prior to immunoprecipitation a chromatin sample of cells treated with either RTEL1 or Poldip3 siRNA were subjected to RnaseH (2U per 100 ul) digest for 30 min at room temperature as controls. Beads were subsequently washed five times in lysis buffer. Cross-link reversal of immunoprecipitated sonicated chromatin were performed by incubating washed beads overnight at 65C in 250 ul of TE buffer containing 1% SDS and 1 ug/ul proteinase K. DNA was purified using PCR purification kit. Q-PCR of each immunoprecipitated sample was performed as described under Q-PCR.

Statistical analyses

Immunofluorescence microscopy quantifications are shown as mean ± SD or percentage and are reproduced in ≥3 independent experiments.

Supplementary references

Sagie S, Toubiana S, Hartono SR, Katzir H, Tzur-Gilat A, Havazelet S, Francastel C, Velasco G, Chedin F, Selig S. 2017. Telomeres in ICF syndrome cells are vulnerable to DNA damage due to elevated DNA:RNA hybrids. *Nat Commun* **8**: 14015.

Schertzer M1, Jouravleva K, Perderiset M, Dingli F, Loew D, Le Guen T, Bardoni B, de Villartay JP, Revy P, Londoño-Vallejo A. 2015. Human regulator of telomere elongation helicase 1 (RTEL1) is required for the nuclear and cytoplasmic trafficking of pre-U2 RNA. Nucleic Acids Res. 43: 1834-47.

Unsal-Kacmaz K, Sancar A. 2004. Quatinary structure of ATR and effects of ATRIP and replication protein A on its DNA binding and kinase activities. Mol Cell Biol 24: 1292-1300.

Urban V, Dobrovolna J, Huhn D, Fryzelkova J, Bartek J, Janscak P. 2016. RECQ5 helicase promotes resolution of conflicts between replication and transcription in human cells. *J Cell Biol* **214**: 401-415.

Wang H, Li S, Zhang H, Wang Y, Hao S, Wu X. 2018. BLM prevents instability of structure-forming DNA sequences at common fragile sites. *PLoS Genet* **14**: e1007816.
